# Differential Impact of Sleep Deprivation and Circadian Timing on Reflexive Versus Inhibitory Control of Attention

**DOI:** 10.1038/s41598-020-63144-y

**Published:** 2020-04-29

**Authors:** Jinny Collet, Suzanne Ftouni, Meaghan Clough, Sean W. Cain, Joanne Fielding, Clare Anderson

**Affiliations:** 1 School of Psychological Sciences and Turner Institute for Brain and Mental Health, Monash University, Clayton, VIC 3800 Australia; 2 Cooperative Research Centre for Alertness, Safety and Productivity, Melbourne, Australia

**Keywords:** Sleep deprivation, Saccades

## Abstract

In a visually stimulating environment with competing stimuli, we continually choose where to allocate attention, and what to ignore. Wake and circadian-dependent modulation of attentional control and resolution of conflict is poorly understood. Twenty-two participants (17males; 25.6 ± 5.6 years) completed ocular motor tasks throughout 40 hours of sleep deprivation under constant routine conditions. A prosaccade task required a reflexive saccade toward a stimulus (no conflict), while an antisaccade task required inhibiting a reflexive saccade to the peripheral stimulus, and looking in the mirror opposite instead (conflict resolution). Antisaccade inhibitory errors showed circadian modulation, being highest in the morning, progressively decreasing until melatonin onset, before returning to the prior morning’s peak throughout the biological night. This diurnal rhythm was blunted by sleep loss (>24 hours), with inhibitory control remaining impaired across the second biological day. For prosaccade, responses slowed down during the biological night. Taken together, we provide evidence for a circadian modulation of attentional bias: the morning being biased toward reflexive responding, and the evening toward higher inhibitory control. Our data show that sleep loss and circadian timing differentially impact attention, depending on whether a response conflict is present (antisaccade) or absent (prosaccade).

## Introduction

The capacity to remain vigilant to the environment, and able to quickly respond to relevant stimuli, is critically important to many modern-day activities, such as driving and surveillance of safety-critical systems. The need for a rapid response to relevant stimuli however, must be balanced against the need to inhibit automatic responses toward distractors (e.g., avoid looking at a flashing billboard while driving). Continual resolution of this conflict, between whether to respond to an external stimulus or to inhibit the response toward it, forms the executive component of attention allocation^1^ and is critical to many higher order cognitive functions^2^.

The wake- and circadian-modulation of sustained attention has been well characterised^3–5^ using the Psychomotor Vigilance Task. In healthy, well-rested individuals performance on this task remains stable across the waking day, followed by a sharp reduction in sustained attention when wakefulness extends beyond 16 hours awake^5,6^. This is due to a combination of both wake (e.g., extended time awake without sleep) and circadian factors (e.g., the drive for sleep during the biological night). While this provides information regarding the capacity to continually monitor and respond to a single stimulus, less is known about wake- and circadian-dependent changes in the resolution of conflict when faced with competing demands, or more specifically on attentional inhibitory control.

From an evolutionary perspective, the circadian regulation of attentional inhibitory control is not only plausible, but advantageous. For instance, an optimal balance between respond (e.g., reactive behaviours in immediate response to danger) and inhibit (e.g., minimising responses to distractors or avoiding aggressive conflict) is clearly beneficial to survival^7^. As evolution has fine-tuned the internal biological clock to best respond to environmental cues to promote survival^8^, the resolution of a respond versus inhibit conflict would be best organised by the internal biological clock.

While inhibitory control has been examined in sleep and circadian studies, these have utilised non-specific, neuropsychological tasks such as the STROOP^9^ or Go/NoGo test paradigms^10^. Moreover, wake-dependent effects are most typically described. For those that have examined circadian-dependent changes in inhibitory control, these studies have employed the STROOP task^9^, which is designed to measure cognitive interference^11^, rather than inhibitory control per se^12^. Moreover the STROOP task is non-specific in that it involves multiple cognitive domains, such as attention, processing speed, cognitive flexibility, and working memory^13^, which is reflected by the recruitment of a vast array of neural circuitry beyond those networks involved in inhibition^14^. Despite these shortcomings, the available literature provides evidence that attentional inhibitory control may be by modulated by the circadian clock. For instance, Burke *et al*. (2015) demonstrated that STROOP performance was highest at the end of the biological day, and lowest after the end of the biological night^9^. While this provides key evidence of our hypothesis, the measure of inhibition used by Burke and colleagues was speed-based [(specifically the difference between congruent (facilitation) and incongruent (interference) median reaction times for correct trials] which represents the cost of inhibition, rather than response inhibition accuracy; this is best captured in terms of the number of inhibition failures, such as error rate for incongruent trials^13^.

We, and others, have utilised the antisaccade task to measure attentional inhibitory control with different clinical disorders and/or under different task conditions^15–19^. The task requires the participant to inhibit a reflexive response toward a peripheral visual stimulus, and instead direct their gaze to the mirror opposite location. The anti-saccade has been widely used to assess cognitive control in clinical populations, given that the neural correlates of these saccadic movements are well understood. As other inhibitory tasks rely on other motor responses, such as a button press, the antisaccade task is proposed as a more selective, independent measure of attentional inhibitory control^17^.

The inhibition of the reflexive response (looking toward the peripheral stimulus) and the allocation of attention elsewhere (to the mirror opposite location) largely depend upon the integrity of the dorsolateral prefrontal cortex and frontoparietal attention networks^20^. As attentional inhibitory control requires successful cooperation between multiple networks known to be vulnerable to sleep loss^21–23^, we hypothesise that the resolution of the attentional conflict between responding to relevant stimuli versus inhibiting responses to irrelevant stimuli will show wake-dependent changes. The extent to which this is modulated by circadian timing remains unknown, although based on evidence from studies on behavioural inhibition/interference, we hypothesise that attentional inhibitory control will be modulated by circadian timing^9^. We therefore examined how the executive control of attention, specifically attentional inhibitory control, is modulated by circadian time and time awake, using an ocular motor antisaccade paradigm.

## Results

**Figure 1:**
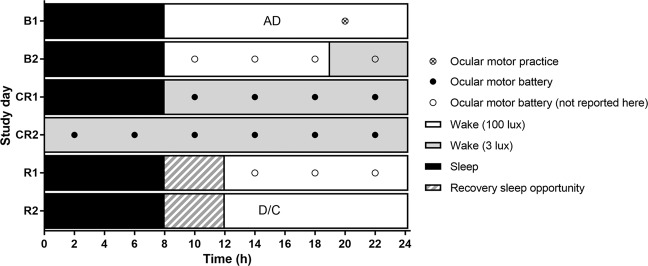
Raster plot of the 6-day laboratory protocol comprising two baseline days (study days B1 and B2), AD indicating admit time, 40 hours of sleep deprivation under constant routine conditions (study days CR1 and CR2) and two recovery days (study days R1 and R2) with up to 12-hour sleep opportunities, before discharge (D/C) from the laboratory. Symbols indicate the timing of tests: Crossed circle (⊗) represents the ocular motor battery practice; black circles (•) represent ocular motor batteries completed during the constant routine; white circles (○) represent additional ocular motor batteries conducted on the baseline and recovery days that are not reported here. White bars represent wake episodes in 100 lux, black bars represent sleep episodes in 0 lux, and grey bars represent <3 lux ambient light for a salivary dim light melatonin onset assessment on B2 and for the 40 h constant routine protocol.

Data were collected from 22 participants across 40 h of total sleep deprivation under constant routine^24^ conditions; see Fig. 1 for in-laboratory study protocol. Two ocular motor tasks were used that tapped into reflexive attention (prosaccade) and inhibitory control (antisaccade). Out of a possible 220 test sessions for each task, we collected data on 212 antisaccade and 211 prosaccade test sessions, representing 3.6% and 4.1% missing data, respectively. Reasons for lost data were due to competing demands within the protocol (e.g., need for IV cannulation), and one participant exiting the study prematurely (at ~34 hours awake).

Since we were primarily interested in attentional control in the absence of sleep events, we separated trials which were confounded with a known ocular index of sleep initiation (e.g., prolonged eye closure) from those without. Test sessions with more than 60% of trials including a sleep-related event were also separated from attention analyses [resulting in the removal of 27 (12.7%) antisaccade and 18 (8.5%) prosaccade test sessions]. Separating these trials allowed us to isolate cognitive processes in relation to inhibitory control from sleepiness or sleep onset events.

**Figure 2:**
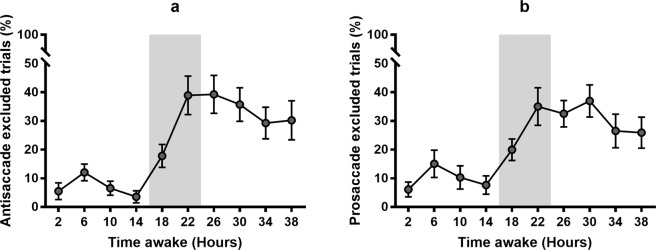
Mean percentage of excluded trials across 40 hours of total sleep deprivation under constant routine conditions. Examples include trials showing ocular indicators of sleep loss such as eye closures, blinks or eye not looking at central fixation for stimulus onset. (**a)** Antisaccade task. (**b)** Prosaccade task. Shaded areas indicate habitual sleep timing during the study protocol. Mean±SEM shown.

Consistent with previous findings in the field^25,26^, sleep deprivation and the biological night were associated with an increased ocular index of sleep initiation. Across all trials, an average of 8.3% (range: 3.55% – 15.0%) of trials were associated with a sleep-related event when well-rested, compared to 30.7% following sleep deprivation (range: 17.8% − 39.3%). Trials associated with ocular indices of sleep initiation significantly increased with time awake for both antisaccade (F_9,140_ = 5.93, P < 0.001) and prosaccade (F_9,180_ = 8.34, P < 0.001) tasks. See Fig. 2.

### Wake dependent effects on attentional control – the influence of time awake

See Table 1 for descriptive statistics.

**Table 1:** Descriptive statistics for the Antisaccade task (Response latency for correct responses and percentage of Antisaccade errors) and the Prosaccade task (Response latency) at each time point relative to time awake (upper panel) or circadian time (lower panel). Mean±SD shown.

	Hours since wake/DLMO	Antisaccade Errors (% trials valid)	Antisaccade Latency (milliseconds)	Prosaccade Latency (milliseconds)
Wake Dependent	2	28.0 ± 19.5	281.8 ± 26.4	171.4 ± 23.0
	6	27.8 ± 16.9	278.8 ± 29.0	174.7 ± 22.3
	10	18.7 ± 16.6	276.8 ± 31.0	178.8 ± 27.6
	14	14.8 ± 14.5	287.8 ± 33.2	177.7 ± 20.1
	18	22.2 ± 11.7	287.2 ± 27.3	189.6 ± 25.5
	22	25.9 ± 11.9	287.8 ± 22.8	188.3 ± 25.4
	26	34.8 ± 16.1	284.5 ± 21.6	187.7 ± 23.9^***^
	30	28.8 ± 16.4	280.5 ± 22.8	185.2 ± 23.0
	34	31.6 ± 19.2^*^	286.1 ± 33.9	184.9 ± 28.4
	38	28.0 ± 19.9^*^	288.6 ± 26.6	183.7 ± 22.9
Circadian Dependent	−12	28.6 ± 19.8^***^	284.5 ± 23.9	170.6 ± 23.3
	−8	27.2 ± 17.0^***^	278.3 ± 29.8	175.2 ± 21.9
	−4	19.5 ± 16.7^*^	277.9 ± 29.1	178.9 ± 28.3
	0	14.3 ± 14.4	286.7 ± 35.2	177.1 ± 19.9
	4	21.4 ± 11.2^**^	286.5 ± 28.4	189.6 ± 25.5^**^
	8	26.7 ± 14.6^***^	285.3 ± 24.0	190.1 ± 24.1
	Biological Day	24.3 ± 16.0	279.3 ± 26.5	175.3 ± 20.8
	Biological Night	21.1 ± 11.9	287.6 ± 27.2	184.8 ± 19.8^***^

Wake Dependent: Significant effects relative to circadian-matched sessions 24 hours earlier (i.e., 2 h vs 26 h awake, 6 h vs 30 h awake, 10 h vs 34 h awake and 14 h vs 38 h awake).

Circadian Dependent: Significant effects relative to dim light melatonin onset (DLMO; hour 0).

*p < 0.05, **p < 0.01, ***p < 0.001.

#### Antisaccade task

**Figure 3:**
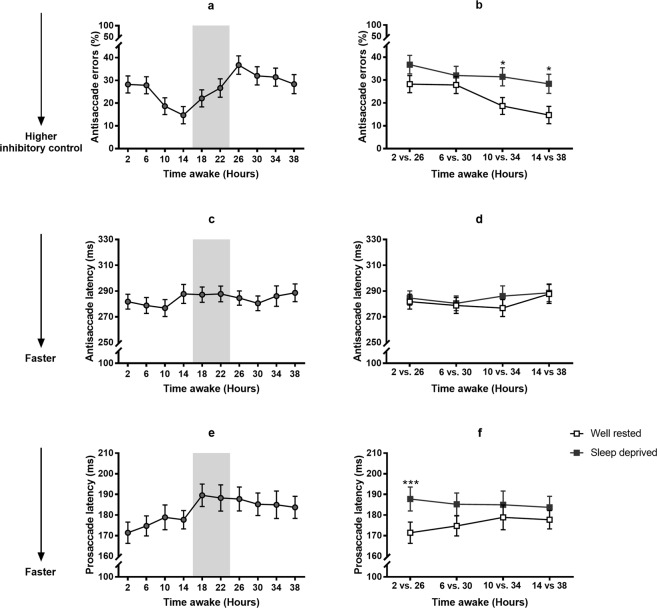
Antisaccade errors, Antisaccade response latency, and Prosaccade response latency with increasing time awake across 40 hours of total sleep deprivation under constant routine conditions. (**a)** % antisaccade errors. (**b)** Comparing % antisaccade errors in the well-rested state versus the sleep-deprived state at matched circadian time points, i.e., 2 versus 26 hours, 6 versus 30 hours, 10 vs 34 hours and 14 versus 38 hours awake. (**c)** Antisaccade response latency (milliseconds). (**d)** Comparing antisaccade response latency (milliseconds) in the well-rested state versus the sleep-deprived state at matched circadian time points, i.e., 2 versus 26 hours, 6 versus 30 hours, 10 versus 34 hours and 14 versus 38 hours awake. (**e)** Prosaccade response latency (milliseconds). (**f)** Comparing prosaccade response latency (milliseconds) in the well-rested state versus the sleep-deprived state at matched circadian time points, i.e., 2 versus 26 hours, 6 versus 30 hours, 10 versus 34 hours and 14 versus 38 hours awake. Note. Shaded areas indicate habitual sleep timing sleep during the study protocol. Mean±SEM shown. *p < 0.05, **p < 0.01, ***p < 0.001.

The percentage of inhibitory errors (i.e., saccades toward the stimulus instead of mirror opposite) increased with time awake (F_9,127_ = 3.97, P < 0.001), reflecting a decrease in inhibitory control (see Fig. 3a). To further examine the effect of sleep deprivation, we compared performance at 26, 30, 34 and 38 hours awake with the corresponding circadian-time matched, well-rested control (2 *vs*. 26 h, 6 *vs*. 30 h, 10 *vs*. 34 h and 14 *vs*. 38 h awake). As seen in Fig. 3b, when controlling for circadian time, we only observed increased errors after 34 hours awake (P_adj_ = 0.02), with no impact of sleep deprivation at either 26 or 30 hours awake (relative to 2 and 6 h awake, P_adj_ > 0.07). Antisaccade response latency on correct trials (the time between peripheral stimulus onset and the initiation of a mirror opposite eye movement) did not change with time awake (P = 0.26, see Fig. 3c,d).

#### Prosaccade task

Prosaccade response latency (i.e., the time between peripheral stimulus onset and the initiation of a saccade toward the stimulus in response) changed significantly with time awake, (F_9,160_ = 6.22, P < 0.0001), becoming slower over the course of the 40 hours of wakefulness (see Fig. 3e). While prosaccade responses were slower at 26 hours compared to 2 hours awake (P_adj_ <0.0001), there were no differences in response speed at 30, 34 and 38 hours awake when compared with the circadian time-matched controls (all P_adj_ > 0.05). See Fig. 3f.

### Circadian dependent effects – the influence of biological timing

Refer to Table 1 for descriptive statistics.

#### Antisaccade task

**Figure 4:**
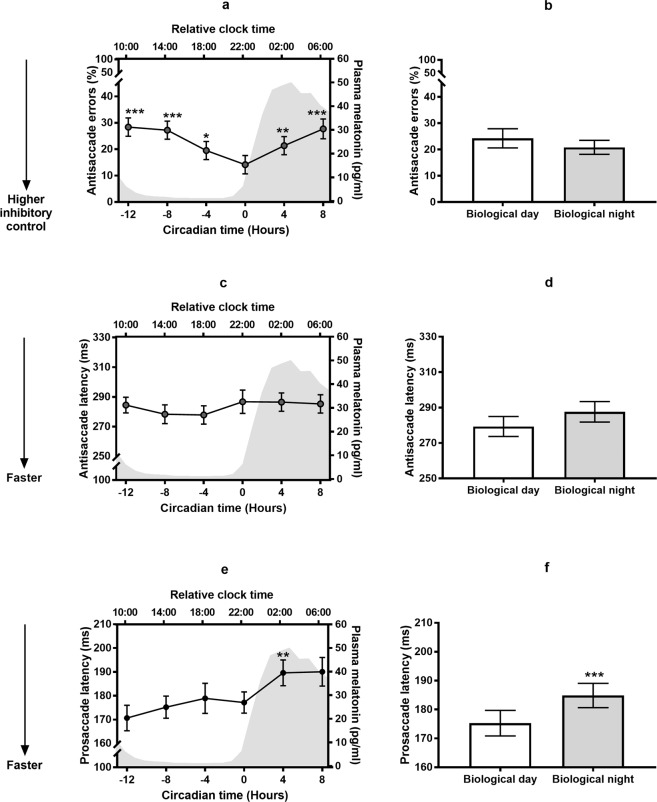
Antisaccade errors, Antisaccade response latency, and Prosaccade response latency relative to circadian time, estimated from plasma melatonin, during the first 24 hours of a 40-hour total sleep deprivation under constant routine protocol. Post-hoc comparisons are relative to the timing of dim light melatonin onset (defined above as hour 0; marking the transition between the biological day and the biological night). (**a)** % antisaccade errors across circadian time. (**b)** Comparing % antisaccade errors in the biological day (−14 to 0 hour relative to dim light melatonin onset) versus the biological night (0 to +10 hours relative to dim light melatonin onset). (**c)** Antisaccade response latency (milliseconds) across circadian time. (**d)** Comparing antisaccade response latency (milliseconds) in the biological day (−14 to 0 hour relative to dim light melatonin onset) versus the biological night (0 to +10 hours relative to dim light melatonin onset). (**e)** Prosaccade response latency (milliseconds) across circadian time. (**f)** Comparing prosaccade response latency (milliseconds) in the biological day (−14 to 0 hour relative to dim light melatonin onset) versus the biological night (0 to +10 hours relative to dim light melatonin onset). Mean±SEM shown. Inlays show melatonin concentration in plasma. *p < 0.05, **p < 0.01, ***p < 0.001.

The percentage of antisacccade inhibitory errors changed as a function of circadian timing (F_5,88_ = 5.18, P < 0.001). As a follow up, we compared each time point relative to dim light melatonin onset, which marks the transition between the biological day (defined as the 14 hours prior to dim light melatonin onset) and the biological night (defined as 0 to +10 hours post dim light melatonin onset). As seen in Fig. 4a, we observed an inverse trajectory whereby the percentage of antisaccade errors decreased across the biological day relative to dim light melatonin onset (P_adj_ = 0.001–0.03 for all time points during the biological day), before rising again throughout the biological night (P_adj_ <0.01). The lowest error rate was thus observed when the task was administered closest to dim light melatonin onset. Given this inverse rhythm, there was no significant mean change in antisaccade errors when comparing the biological day to the biological night (P = 0.27, *d* = 0.25; see Fig. 4b). As seen for time awake, antisaccade response latency on correct antisaccade trials did not change with circadian timing, despite changes in error rate (P = 0.17, See Fig. 4c,d).

#### Prosaccade task

Prosaccade response latency differed across circadian time of day (F_5,93_ = 7.02, P < 0.0001; see Fig. 4e) such that responses were, on average, slower during the biological night compared to the biological day (*t*(21) = 4.49, P_adj._ <0.0001, with a large effect size *d* = 0.96; see Fig. 4f). Responses were slower during the biological night at 4 hours post-dim light melatonin onset (P_adj_ <0.01), and trending toward significance at 8 hours post-dim light melatonin onset (P_adj_ = 0.06). No significant differences were observed for any time point during the biological day relative to dim light melatonin onset (all P_adj_ > 0.1; see Fig. 4e).

## Discussion

Our study provides evidence that inhibitory control is modulated by the circadian timing system, and that sleep loss and circadian time differentially impact attentional control, depending on whether or not a response conflict is present. Our novel data suggest circadian modulation of attentional bias, whereby the morning is biased toward reflexive responding, while the evening is biased toward higher inhibitory control. This has important ramification for both the evolutionary development and organisation of the attention system, but also the management of attention failures in the modern workplace.

Conceptually, the antisaccade task presents a respond versus inhibit conflict; the conflict being the choice between the bottom up response (a reflexive saccade toward the peripheral stimulus) versus the top down controlled response (inhibit the reflexive saccade, look at the mirror opposite location). Inhibitory errors following sleep deprivation were not observed at either 26 or 30 hours awake, but did increase after 34 hours awake when controlling for circadian time. This is consistent with previous work utilising a single time point in the early-mid afternoon^16,27^, and is likely caused by inadequate top-down inhibitory control from the dorsal frontoparietal network^20,28^. Our study is unique in providing the first evidence of the circadian modulation of conflict resolution within the attention system. Here, antisaccade inhibitory errors were highest in the morning, decreased across the biological day until the beginning of the biological night (i.e., ~dim light melatonin onset), before rising back to the prior morning’s peak in errors throughout the biological night. This observation is consistent with previous findings showing a reduced cost of inhibition in the early evening using the STROOP task^9^.

While this improvement in inhibitory control across the course of the biological day may appear to be a practice effect or dissipation of sleep inertia, we argue that this is unlikely. Firstly, our participants were exposed to>200 trials prior to our tests during the constant routine, which is more than the 10 trials recommended to remove practice effects^29^. Moreover, there was no difference between test 1 and test 5 (time matched) examined prior to the constant routine protocol suggesting a removal of the practice effects (see Methods). Secondly, sleep inertia typically lasts for <4 hours^30^, and improvements were not observed for speed of responses on either task. Instead, we speculate that our results may reflect wake and circadian changes in arousal. For instance, heightened arousal is known to facilitate the reflexive response, leading to an increase in antisaccade errors^31^. The biological clock temporally regulates arousal^32^ via the locus coeruleus norepinephrine system, whose signals are proposed to enable transitions between sleep (low arousal), focused alert (moderate arousal), and exploratory (high arousal) states^33^. Using this framework, optimal task focus (i.e., inhibitory control) should be observed when arousal is moderate rather than when arousal is high, as per the morning hours, or low, as per sleep deprivation. While our data do support this interpretation, this remains speculative and future studies examining the simultaneous modulation of arousal (e.g., pupillography) would be required.

The wake and circadian modulation of prosaccade response times were consistent with the widely examined psychomotor vigilance task: slower response time with increasing sleep loss, with a partial recovery observed on day 2 when the circadian system is promoting wakefulness^3,5,25,26^. This replication is important because the prosaccade task is similar to the psychomotor vigilance task in that both tasks require individuals to monitor and quickly respond to a single stimulus in the absence of conflicting external demands on the attention system^4^. In contrast, antisaccade response times did not change with circadian timing or time awake on trials when the attentional conflict was correctly resolved (i.e., not errors). While this may appear counterintuitive (i.e., no change in response time with increasing sleep loss), it is consistent with the notion of state instability, whereby at the trial-to-trial level, the brain can switch from impaired (i.e., an error) to intact (i.e., a correct antisaccade response with unchanged speed). Future neuroimaging work is required however to investigate the neural correlates of circadian and wake-dependent changes during the antisaccade and prosaccade tasks to understand the mechanisms behind trial by trial variability.

A key strength of our study is the use of the saccade as an outcome. Firstly, saccades are universal and the most frequent movement during wake^34^, thus eliminating potential confounds due to learned skills. Secondly, by tracking the eyes, we were able to isolate cognitive effects from sleep-related events and therefore focus on decision-related attentional processes, without unintentional sleep onset as a confound. Thirdly, examining one behaviour (the saccade) within the one sensorimotor system (the ocular motor system) eliminates confounds that might arise from tasks using different sensorimotor modalities (e.g., button presses). Fourthly, and perhaps most important, the antisaccade offers advantages over other typically used measures of inhibition involving multiple cognitive domains (e.g., STROOP), allowing for a more selective examination of attentional control^17^. Finally, as our two tasks are identical at the behavioural level (both require an eye movement toward a peripheral location), we are able to examine how sleep and circadian timing impact low-level (bottom up, stimulus-driven) and high-level (top down, goal-directed) attentional control in the absence of many task confounds.

Our data also have some limitations and should therefore be interpreted with the following caveats in mind. Firstly, we conducted the study in a sample of healthy, young adults, thus the findings may not generalise to older individuals, or to those with underlying brain pathology, particularly since the antisaccade response is impaired in older adults^35^ and in psychiatric patients^36^. Secondly, we are not fully able to isolate wake- and circadian-modulation of attentional control due to employing a constant routine protocol, rather than a forced desynchrony [e.g.,^9^]. Thirdly, our study findings may not replicate under conditions of chronic, partial sleep loss, and future work should address this given the importance of attentional control in real-world settings.

To summarise, in relation to attentional control, when no conflict resolution is required (prosaccade task), our data are consistent with that previously reported for sustained attention. Since we live and work in environments that are rarely free of distractors, it is important to consider the allocation of attention when faced with conflicting demands. In this instance, attention appears biased toward reflexive (bottom up) responding in the morning, with inhibitory (top down) control being highest at the end of the biological day/start of the biological night. Why this attentional selection preference has evolved and how it may signify adaptation to changes in predatory danger, food availability, or offspring risk is unknown, although the concept of the evolution of the attention system as a function of the 24 light/dark cycle is intriguing. In today’s environment though, the extent to which we fail to resolve attentional conflict in the morning compared to the evening in real environments is unknown. These questions concerning the circadian modulation of attentional control remain important avenues for future research.

## Methods

### Participants

Twenty-two healthy adults (17 males, 5 females; mean age ± SD = 25.60 ± 5.62 years; range 20–45 years) took part in the study at the Monash Sleep and Circadian Medicine Laboratory. Participants were screened to ensure no physical or psychological disorders, as verified via medical and clinical psychologist assessment, respectively. Any individuals who reported a family history of psychiatric illnesses within their first degree relatives were also excluded. Women were not currently pregnant (confirmed with urine test) or using hormonal contraception, and the study was conducted during the follicular phase of their menstrual cycle to control for any impact of menstrual phase on performance^37^. All participants had normal colour vision as determined by the Ishihara colour-blindness test^38^. Participants reported sleeping between 7 and 9 hours per night, napping no more than once per week, and were free from any sleep disorders as confirmed using full polysomnography. They also reported no complaints about sleep quality [<6 on the Pittsburgh Sleep Quality Index^39^]; or daytime sleepiness [<10 on the Epworth Sleepiness Scale^40^]. They had not travelled across three time zones or more in the past month or undertaken shift work (5 or more hours worked between 10:00 pm and 07:00 am) within the past 3 months. Participants had a body mass index between 18.0 and 29.9 kg/m^3^, reported no use of illicit substances within the past year, and did not consume more than 300 mg per day of caffeine, or more than 14 standard units per week of alcohol. The study was approved by the Monash University Human Research Ethics Committee, reference CF14/1765–2014000877. All participants provided written informed consent prior to the study, and the study was conducted in accordance with the Declaration of Helsinki.

### Pre-laboratory study procedures

The pre-laboratory study procedures are summarised here, having been previously reported elsewhere^41,42^. For 2 weeks prior to laboratory admission, participants maintained a structured 8:16 hour sleep:wake schedule, confirmed via wrist-worn actigraphy (Philips Respironics, BMedical, Australia), sleep diaries and calls to a time- and date- stamped voicemail at sleep and wake times. Throughout the study, participants refrained from use of any prescription or non-prescription medications, supplements, recreational drugs, caffeine, alcohol, or nicotine. Compliance was verified with urine toxicology and breathalyser assessment upon laboratory admission.

### In-laboratory protocol

Participants were studied for 6 days in a private, sound-attenuated room free of time cues. Their schedule consisted of a 2-day baseline with the same habitual 8:16 h sleep:wake cycle as that maintained over the 2 weeks prior to laboratory admission. This was followed by a 40 hour constant routine protocol^24^ and two extended 12-hour sleep recovery opportunities prior to discharge. See Fig. 1 for protocol illustration. Light levels were strictly controlled throughout the study: typical room lighting during baseline and recovery wake episodes was 100 lux (102 ± 37 lux in the horizontal plane and 45 ± 21 lux in the vertical plane), and <3 lux during the constant routine protocol (3 ± 1 lux in the horizontal plane and 1 ± 3 lux in the vertical plane). Lights were turned off for sleep. Lighting was generated from ceiling-mounted 4100 K fluorescent bulbs (Master TL5 HE 28 W/840 cool lights, Philips Lighting, Amsterdam, Netherlands), covered with neutral density filters (3-stop LEE Filters, Lightmoves, Noble Park, Australia), which provided broad-spectrum white light with a peak of 545 nm and CCT of 3968 K (UPRTek MK350N Spectrometer, Taiwan). All data reported here were collected during the constant routine protocol, when participants remained awake under constant dim light (<1 lux at the vertical and <3 lux horizontal plane). Temperature was constant (21 °C ± 2 °C) and participants remained in a semi-recumbent 45° posture throughout. Daily nutritional intake was served in hourly portions of isocaloric meals (quarter sandwich, 60 ml water, 40 ml juice), and participants were continually supervised by study staff during the constant routine protocol to ensure continued wakefulness and compliance.

### Melatonin measurement and radioimmunoassay

Plasma melatonin was used as the marker of circadian timing^43^. Blood samples were collected at hourly intervals using an indwelling intravenous cannula, inserted into the forearm or antecubital vein within approximately one hour of the participants waking onto the constant routine protocol. At each collection, whole blood was collected in a syringe and aliquoted into a blood tube spray coated with K2EDTA. Samples were immediately centrifuged at 4 °C and spun at 1,300 x g for 10 minutes to derive plasma samples (1 ml) which were snap-frozen in dry ice, and stored at -20°C until assayed. Total plasma melatonin was determined at the Adelaide Research Assay Facility by reverse-phase C-18 column extraction of 500 µl plasma^44^, followed by double antibody radioimmunoassay using standards and reagents supplied by Buhlmann Laboratories (RKMEL-2, Buhlmann Laboratories AG, Schönenbuch, Switzerland). This assay is based on the Kennaway G280 anti-melatonin antibody^45^ and [125I]2-iodomelatonin as the radioligand. This follows the protocol provided by Buhlmann. The sensitivity of the assay using 500 µl of extracted plasma was 1.0 pg/ml. Plasma samples were assayed in four separate batches and in duplicate. All samples from an individual were measured in a single assay. The intra-assay coefficient of variation was 5.5–9%. The inter-assay coefficient of variation of the low concentration quality control was 5.3–13.3%, and the inter-assay coefficient of variation of the high concentration quality control was 7.3–17.7%.

### Measuring attentional control: ocular motor test paradigms

We used ocular motor paradigms to examine changes in attentional control due to extended time awake and circadian timing. Our primary task was the antisaccade task where participants are required to inhibit the reflexive saccade toward a peripheral stimulus and look mirror opposite instead, measuring top down inhibitory control^15^. Participants also completed the prosaccade task as a secondary task outcome. This involved no conflict resolution and required participants to make a reflexive saccade toward the peripheral stimulus. We utilised the prosaccade task as it mimics the Psychomotor Vigilance Task (on which much of the sleep/circadian attention literature is based) in that both tasks require a response to a single recurring stimulus in the absence of any external conflicting demands on the attention system). This allows us to compare our results to previous literature, but is more appropriate to compare against the antisaccade task in that both tasks use the same sensory cue (visual stimulus) and require the same motor response (saccade).

During the ocular motor tasks, participants’ eye movements (saccades) are monitored via an eye tracker. Monocular eye movements were recorded at a sampling rate of 1000 Hz, using an Eyelink 1000 Plus eye tracker (SR Research Ltd, Ontario, Canada). The stimulus consisted of a green cross subtending 1.6° × 1.6° of visual angle, presented against a black background. Each trial began with participants staring at the stimulus at central fixation. The stimulus then appeared in a peripheral location (5° or 10° to the left or right, at random but equal frequency), while simultaneously being extinguished from central fixation (no gap). The visual stimulus was then extinguished at the periphery and simultaneously reappeared at central fixation in preparation for the next trial.

Participants completed five test sessions prior to the Constant Routine: one full ocular motor test battery after admission to the laboratory on day 1, followed by four administrations on day 2. Data presented here are derived from ocular motor tasks administered throughout the constant routine protocol (days 3 to 4) at 4-hourly intervals, beginning 2 hours post-wake. Task order was randomised between test sessions. As a check on practice effects, we compared the first task administration with the fifth (time matched) and found no significant differences.

#### Antisaccade task

After staring at the central fixation for 1500 ms, participants were required to inhibit the automatic prosaccade response toward the peripheral stimulus, and instead initiate a horizontal saccade to the mirror opposite location^15^. The peripheral stimulus remained on-screen for a random duration of 1250 or 1600 ms. Trials where participants looked toward the stimulus instead of the mirror location were classified as antisaccade inhibitory errors. Percentage antisaccade errors was calculated as a percentage of valid trials (not total trials), reflecting changes in inhibitory control when participants’ eyes were open and when they were able to respond to the stimulus. Antisaccade response latency was calculated for correct antisaccade response, defined as the time between peripheral stimulus onset and the initiation of a saccade to the mirror opposite location in response. Two successive blocks of 24 trials were presented resulting in a total task duration approximating 180 seconds.

#### Prosaccade task

After staring at the central fixation for a random duration between 1000, 1250 and 1500 ms, participants were required to initiate a horizontal saccade as quickly as possible toward the peripheral stimulus. Prosaccade response latency was defined as the time elapsed between the appearance of the peripheral stimulus and the initiation of a saccade towards it. One block of 24 trials was presented resulting in a total task duration approximating 73 seconds.

### Data analysis

#### Dim light melatonin onset

Dim light melatonin onset was calculated for each participant^46^ as the time at which the concentration of plasma melatonin crossed the 5 pg/ml threshold as determined by linear interpolation between measured values flanking the threshold^47^ (we have used a low threshold for DLMO to best accommodate individual variation in melatonin secretion^48^, plus data are binned in four-hourly bins). Dim light melatonin onset was assigned a value of 0 hour, and circadian time for each ocular motor battery session across the first 24 hours of the constant routine protocol were expressed relative to dim light melatonin onset.

#### Ocular motor data

Data were analysed using a customised MatLAB program (MATLAB, The Mathworks Inc., Natick, MA). Trials were excluded if the participant’s eye was closed or not within 2.5° of central fixation upon stimulus presentation. Trials were removed for separate analysis if participants blinked or closed their eye between stimulus presentation and the completion of their saccade response, or removed if their response latency was below 100 milliseconds, which was considered an anticipatory eye movement^16^. Data from a test session were excluded if more than 60% of trials were separated for that session. Response latency was transformed to its reciprocal 1/[x/1000] to normalise the data^16^.

#### Statistical analyses

To assess the effect of time awake across the 40 hour constant routine, linear mixed-model analyses (SPSS 24.0, IBM Corp., Armonk, N.Y.) were performed with *Participant* modelled as a random effect and *Time awake* as a fixed effect, using the Kenward-Rogers method to calculate degrees of freedom. Post-hoc pairwise comparisons were conducted on task outcomes 24 hours apart (i.e., circadian time matched control) in the well-rested vs sleep-deprived state; specifically 2 hours versus 26 hours, 6 hours versus 30 hours, 10 hours versus 34 hours, and 14 hours versus 38 hours awake. To assess the effect of circadian phase, task outcomes administered within the first 24 hours of the constant routine protocol were modelled with *Participant* as a random effect and *Time relative to dim light melatonin onset* as a fixed effect. Post-hoc pairwise comparisons were conducted to compare against the task administered closest to the time of dim light melatonin onset, marking the transition between the biological day and the biological night. False discovery rate (FDR) comparisons were used to control for type I error, and adjusted p values are reported using the FDR “*q*” adjusted significance values^49^.

## Data Availability

Raw latency and errors data are available. Requests for further data access will be considered on a case-by-case basis. Applications for data access should be sent to Dr. Clare Anderson (clare.anderson@monash.edu).
